# A MATLAB tool for pathway enrichment using a topology-based pathway regulation score

**DOI:** 10.1186/s12859-014-0358-2

**Published:** 2014-11-04

**Authors:** Maysson Ibrahim, Sabah Jassim, Michael Anthony Cawthorne, Kenneth Langlands

**Affiliations:** Department of Applied Computing, the University of Buckingham, Buckingham, MK18 1EG UK; The Buckingham Institute for Translational Medicine, the University of Buckingham, Buckingham, MK18 1EG UK

**Keywords:** ■■■

## Abstract

**Background:**

Handling the vast amount of gene expression data generated by genome-wide transcriptional profiling techniques is a challenging task, demanding an informed combination of pre-processing, filtering and analysis methods if meaningful biological conclusions are to be drawn. For example, a range of traditional statistical and computational pathway analysis approaches have been used to identify over-represented processes in microarray data derived from various disease states. However, most of these approaches tend not to exploit the full spectrum of gene expression data, or the various relationships and dependencies. Previously, we described a pathway enrichment analysis tool created in MATLAB that yields a Pathway Regulation Score (PRS) by considering signalling pathway topology, and the overrepresentation and magnitude of differentially-expressed genes (*J Comput Biol* 19:563–573, 2012). Herein, we extended this approach to include metabolic pathways, and described the use of a graphical user interface (GUI).

**Results:**

Using input from a variety of microarray platforms and species, users are able to calculate PRS scores, along with a corresponding z-score for comparison. Further pathway significance assessment may be performed to increase confidence in the pathways obtained, and users can view Kyoto Encyclopedia of Genes and Genomes (KEGG) pathway diagrams marked-up to highlight impacted genes.

**Conclusions:**

The PRS tool provides a filter in the isolation of biologically-relevant insights from complex transcriptomic data.

**Electronic supplementary material:**

The online version of this article (doi:10.1186/s12859-014-0358-2) contains supplementary material, which is available to authorized users.

## Background

Increasingly, high-throughput transcriptional profiling techniques (microarrays or, increasingly, RNAseq) inform modern life-science research. Such techniques provide a molecular “camera” taking genome-wide “snap-shots” of genetic activity. However, the effective analysis of microarray data presents a number of challenges, in particular handling the large number of genes that are studied simultaneously.

Analysing gene expression in the context of curated knowledge, or “knowledge base-driven pathway analysis”, is critical as this guides the reduction in search space from many thousands of genes to an subset of biological processes, which are much more tractable to human interpretation [1]. According to Khatri *et al* [2], pathway enrichment approaches can be divided into three generations:

i. Over-representation Analysis (ORA): This scores a pathway by considering the proportion of differentially-expressed genes (DEGs) observed in each pathway relative to the proportion of all microarray DEGs. This is used by several pathway analysis tools, including GenMAPP [3], GoMiner [4], Onto-Express [5] and FatiGo [6].
ii. Functional Class Scoring (FCS): FCS gives a score to each gene in a pathway based on its expression, from which a pathway-score is calculated based on the scores of all the genes in the pathway. A number of FCS methods have been implemented through standalone tools such as GSEA [7], SigPathway [8], and SAFE [9], or web tools such as T-profiler [10], Gazer [11] and GeneTrail [12].
iii. Pathway Topology (PT)-based approaches: These approaches exploit the topology of pathways by giving weights to pre-defined connections between genes, which inform pathway scoring. Several topology-based approaches have been described in the literature over the past few years. According to Mitrea et al [13], PT-based approaches differ in the way they translate pathway topology information into a pathway score. Some methods use only the topology data of differentially-expressed genes (DEGs) in the enrichment score (for example MetaCore [14] and EnrichNet [15]), whereas others (including SPIA [16] and GANPA [17]) use expression data of DEGs along with the topology data. Alternatively, some methods use expression data derived from all microarray genes, whether they change between conditions or not, for example PathOlogist [18], DEGraph [19], and ACST [20]. Importantly, some PT-based tools use only signalling pathway descriptions, such as Pathway-Express [21], NetGSA [22], ScorePAGE [23], TAPPA [24] MetPA [25], and Clipper [26].

Previously, we proposed a new pathway enrichment method, in which both pathway topology and the magnitude of gene expression changes informed the creation of a Pathway Regulation Score (PRS) [27]. Specifically, by combining fold-change data for those transcripts exceeding a significance threshold, and by taking into account the potential of altered gene expression to impact upon downstream transcription, we identified those pathways most relevant to the pathophysiological process under investigation. Our approach addressed a number of issues that potentially compromise enrichment methods. We took steps to mitigate the influence of errors in ID mapping, and to reduce the bias introduced by highly-redundant pathways (i.e. multiple instances of the same gene). Topology methods also have to handle loops effectively, so we used a search algorithm derived from graph theory to resolve this problem. We also felt that arbitrarily dividing processes into either up- or down-regulated was artificial as changes in gene expression are likely to be distributed throughout pathways, thus ours was an overall impact assessment.

Herein, we described the implementation of our PRS approach as a standalone tool that provides end users with the option of importing data from different microarray platforms and species. The tool yields both PRS and z-scores, provides statistical analysis, and allows browsing of pathways with impacted genes highlighted in different colours. An enhancement from our original report is that users are able to enrich both signalling and metabolic pathways.

## Implementation

**Figure 1:**
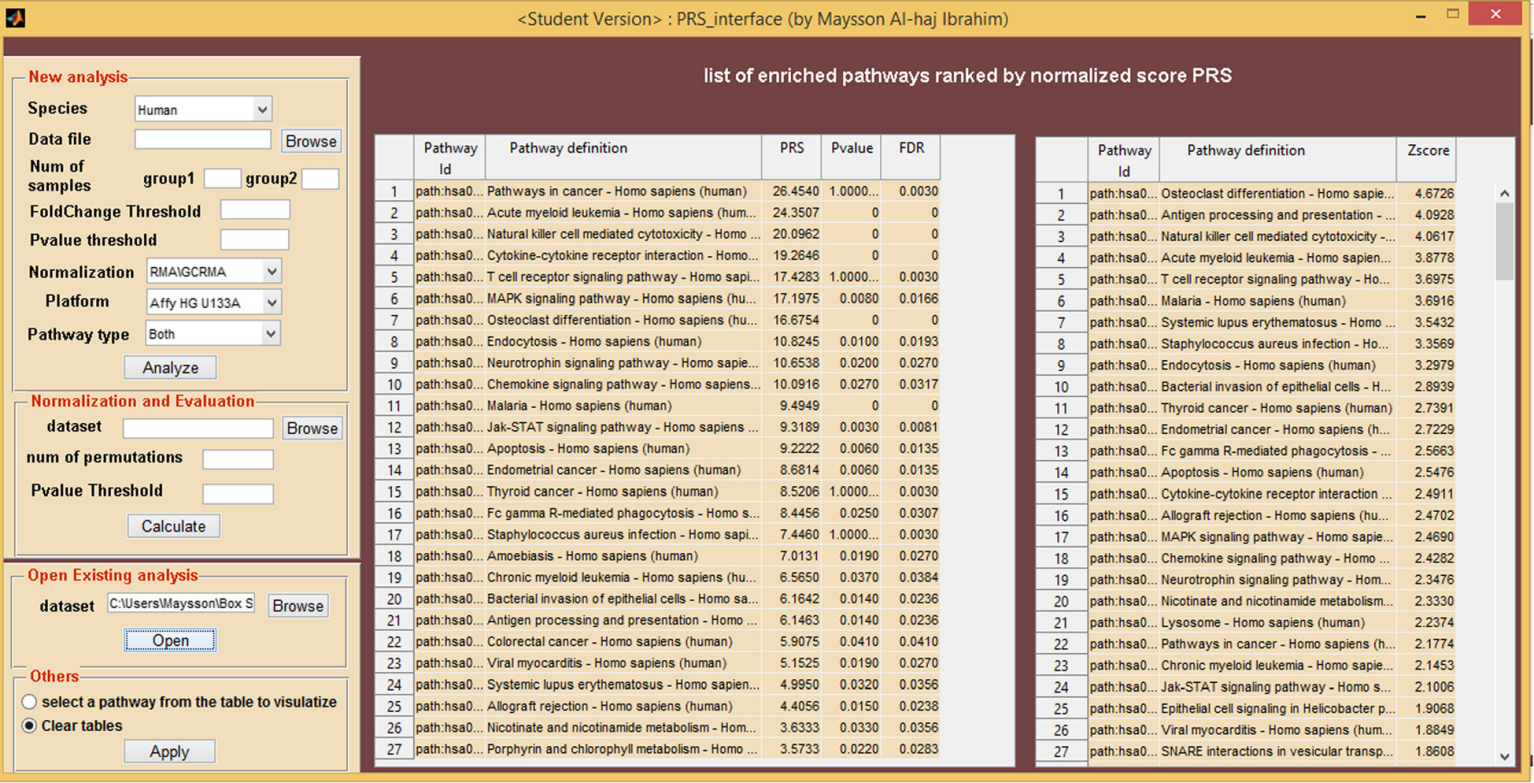
**The PRS user interface showing analysis of a sample dataset.**

The PRS approach was implemented in MATLAB. Users without access to the MATLAB environment can down-load the MATLAB Runtime Compiler (MRC) in order to deploy the software described herein, via a user-friendly GUI. The PRS interface (Figure 1) provides users with several functions:

### Preprocessing microarray data

We did not re-engineer a filter to normalise data from a variety of platforms, rather users must first preprocess transcriptomic data using one of the myriad existing tools. Data must be in the form of a simple Excel spreadsheet, in which the first column should be probe ID, and the following columns normalised replicated expression values from the control and test conditions. Additional information regarding species, sample numbers, fold-change and *t*-test thresholds, normalisation method and platform is required.

### Pathway representation

**Figure 2:**
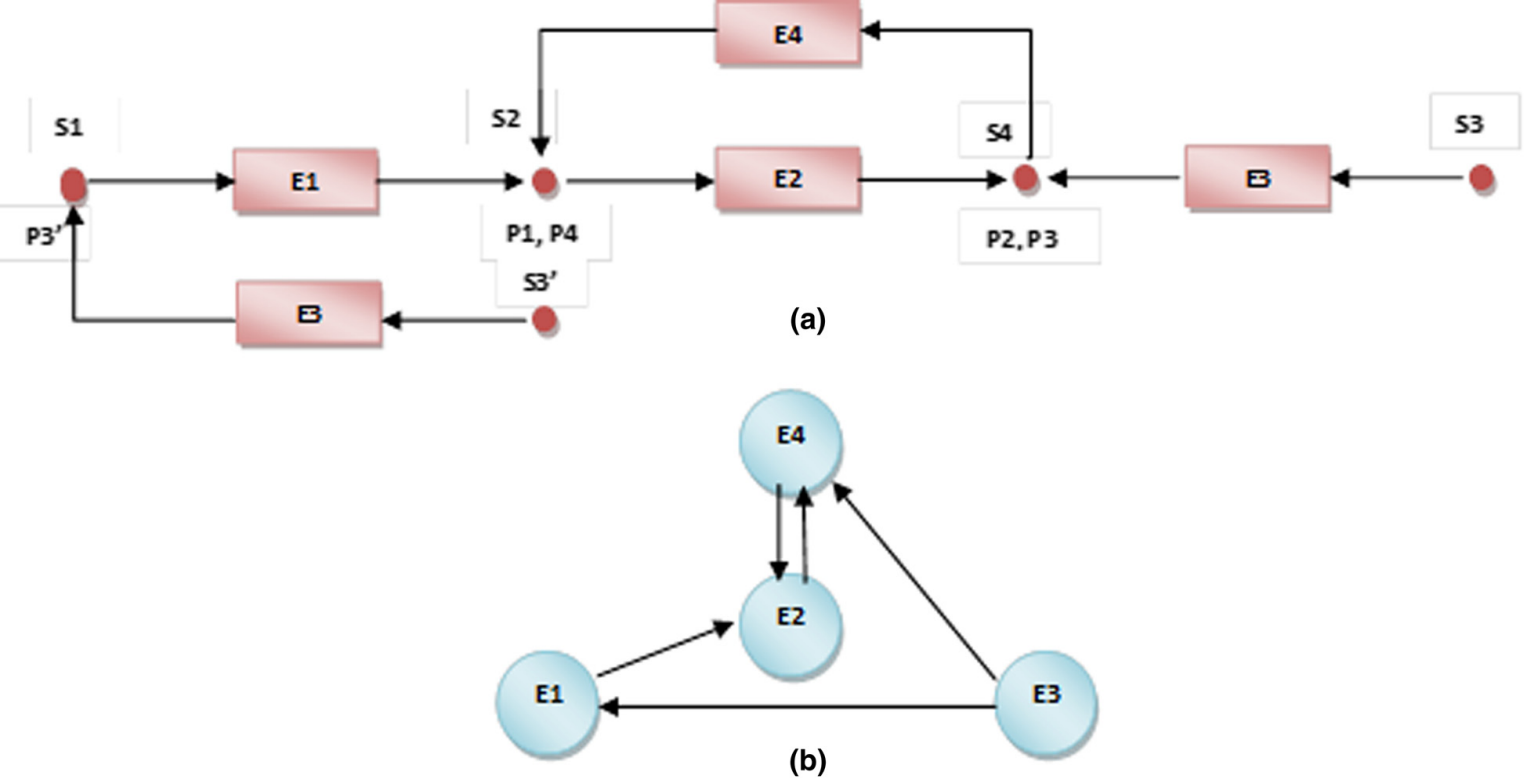
**Example of the conversion of a group of reactions in a metabolic pathway (a) into a diagraph (b) after removing redundancy.**

Our fundamental algorithm was described previously [27]. Briefly, Kyoto Encyclopaedia of Genes and Genomes pathway definitions [28] were used, in which pathways are maintained in KEGG Mark-up Language (KGML) format. We imported a total of 189 signalling and metabolic descriptions from KEGG and parsed these into MATLAB objects, which were then converted into directed graphs. KGML files contain three types of objects: entries, relations, and reactions. These can be mapped to graphical objects in the associated pathway map (Additional file 1). Only entries (which form nodes, represented as boxes) and relations (represented as edges) were used to represent signalling pathways where proteins (boxes) are linked by “relations”. All three types are used to represent the structure of metabolic pathways in order to capture substrate-enzyme-product relationships where enzymes (boxes) are linked by “relations”, and compounds (circles) are linked by “reactions”. To convert a metabolic pathway into a graph in a rational way, we represented enzymes as nodes in the graph, while substrates and products were used to detect the direction of relations (edges) between nodes (Figure 2). While we acknowledge that is not possible to predict any effect on flux by this rationale, we reasoned that any change in node expression in a metabolic pathway could be of physiological relevance, particularly if nodes were connected.

Representing pathways as graphs had an additional advantage as it reduced redundancy in that genes were only represented once in any pathway graph. A Depth-First Search (DFS) algorithm, derived from Graph Theory was used to ensure that loops were only counted once.

### Pathway scoring

Our method assigned weights to all significant nodes (i.e. DEGs) in a pathway to reflect their topological strength (specifically the number of significant downstream nodes that are pointed to, either directly or via other significant nodes as described previously [27]). A PRS was calculated on the basis of fold-change value and weighting of all significant nodes in the pathway and normalized for pathway size. We also calculated a z-score [29] (with an improvement over earlier implementations in that this was performed after removing redundant genes from pathway descriptions). The software outputs two lists of pathways ranked according to PRS and z-score, saved as both Excel and .mat files for later analysis.

### Pathway significance assessment

We then went on to establish the probability of achieving scores at least as high as the PRS score by chance using a non-parametric permutation method. Initially, fold-change values for all expressed microarray genes were permuted. These values were then mapped back onto pathways, and a PRS recalculated. This process was repeated *n* times, where *n* is provided by the user through the interface (typically n = 1000). The statistical significance (p-value) of each pathway score was estimated by a comparison between the observed score and the *n* random scores generated. To achieve more reliable statistical significance evaluation, p-values were adjusted for multiple-test correction by a False Discovery Rate (FDR) method based on a threshold provided by the user. This is described in more detail in our original report [27].

### Visualizing enriched pathways

**Figure 3:**
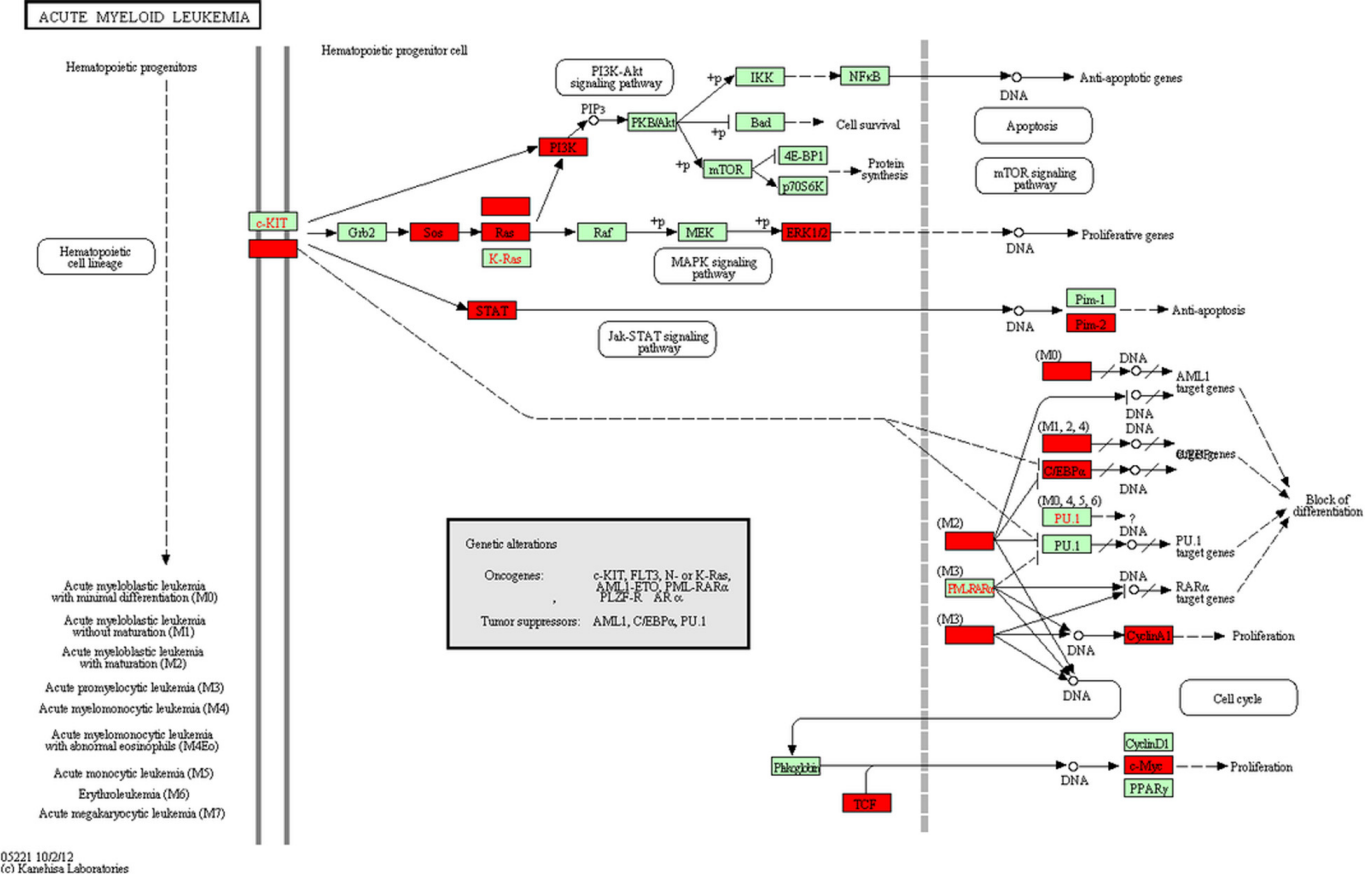
**A typical marked-up pathway, in this case the KEGG “acute myeloid leukaemia pathway” enriched in an AML dataset (GEO accession #GSE9476); significant genes are coloured in red and non-significant ones in green.**

After running the analysis, results are saved as .mat format files for ease of retrieval. By clicking on the pathway name from the list of ranked pathways shown in the table and selecting the option of visualizing a pathway from the interface, a marked-up pathway map will be displayed. Technically, the software will call a pathway mapping web service (REST-based API service) hosted on the KEGG website and pass a number of parameters, including a list of all expressed genes with their fold- changes and specified colours to differentiate DEGs from non-impacted genes. Figure 3 shows a typical pathway map where significant (i.e. above threshold) genes are coloured in red and non-significant (i.e. unchanged or not expressed) in green.

### UML for modelling and software description

**Figure 4:**
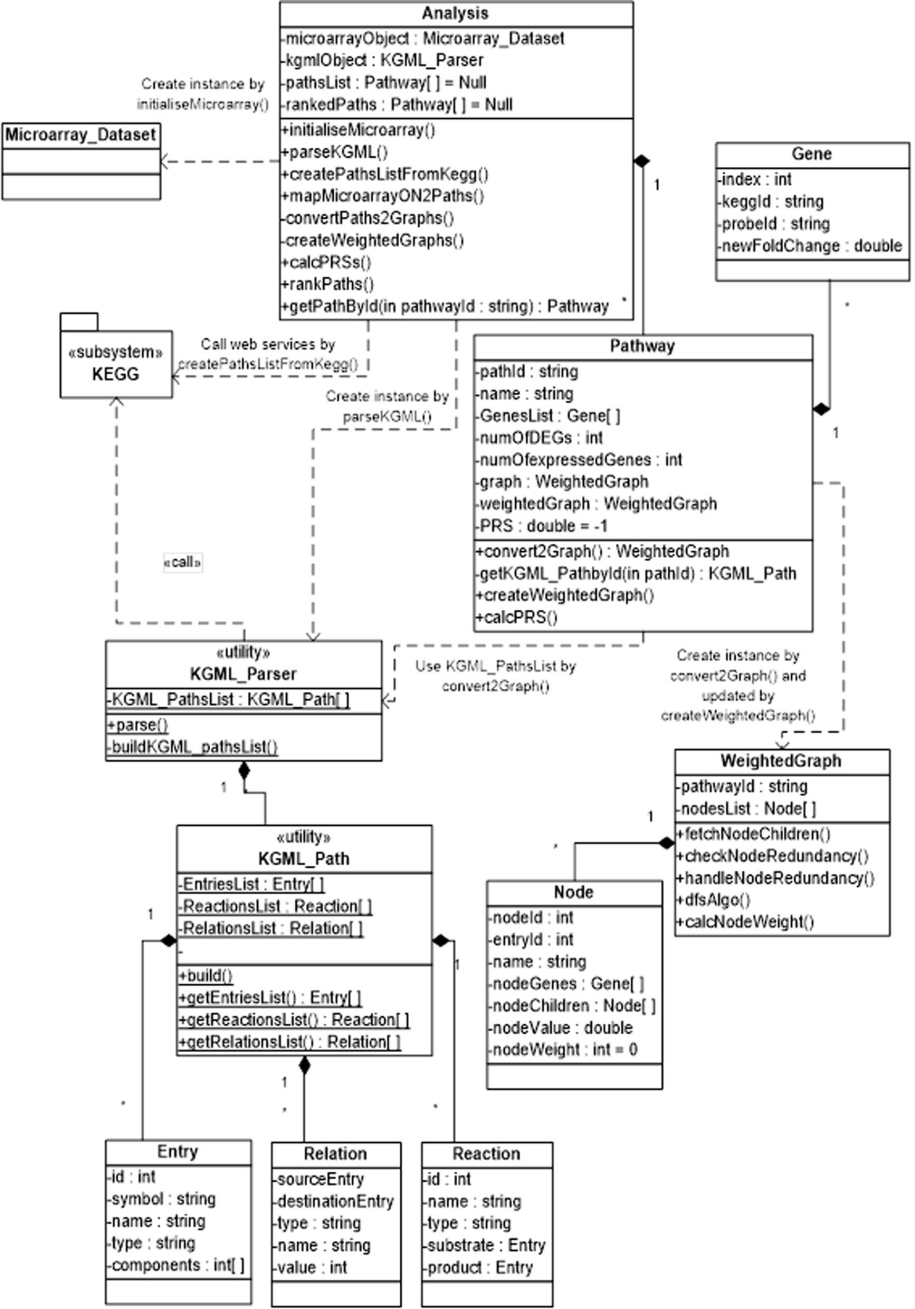
**UML class diagram illustrating the main classes of the package at the pathway analysis stage.**

Herein, we used Unified Modelling Language (UML) to describe, model and visualize the structure and functions of our method by diagrams. There are 14 types of diagrams classified in three categories in UML 2.0 [30], however, in this paper we used only two: class and sequence diagrams. Class diagrams represent static structures or main objects in the software. Figure 4 shows the key classes at the pathway analysis stage. The class “Analysis” is the main class, which provides an interface to run all the services provided by the tool. It has four main attributes:

▪ MicroarrayObject: an object of the class “Microarray_Dataset” built by calling initialiseMicroarray() function (see Additional file 2). This holds the normalised gene expression data, and a list of all genes with their fold-change values.
▪ kgmlObject: an object of the class “KGML_Parser” built by calling the parseKGML() function (see Additional file 3). This holds the static structure of all pathways as a list of objects of “KGML_Path” class that is defined by KGML format. An object of “KGML_Path” represents the structure of one KEGG pathway and is composed of entriesList, reactionsList, and relationsList (see Additional file 1).
▪ PathList: this is a list of objects of the class “Pathway” which is created by calling CreatePathListFromKegg() function (see Additional file 4). This object ultimately holds a list of pathways enriched with reference to a given microarray dataset.
▪ rankedPaths: this object is created by calling the rankPaths() function. It holds the same list of pathways defined by PathsList, but they are ranked in descending order based on PRS values.

**Figure 5:**
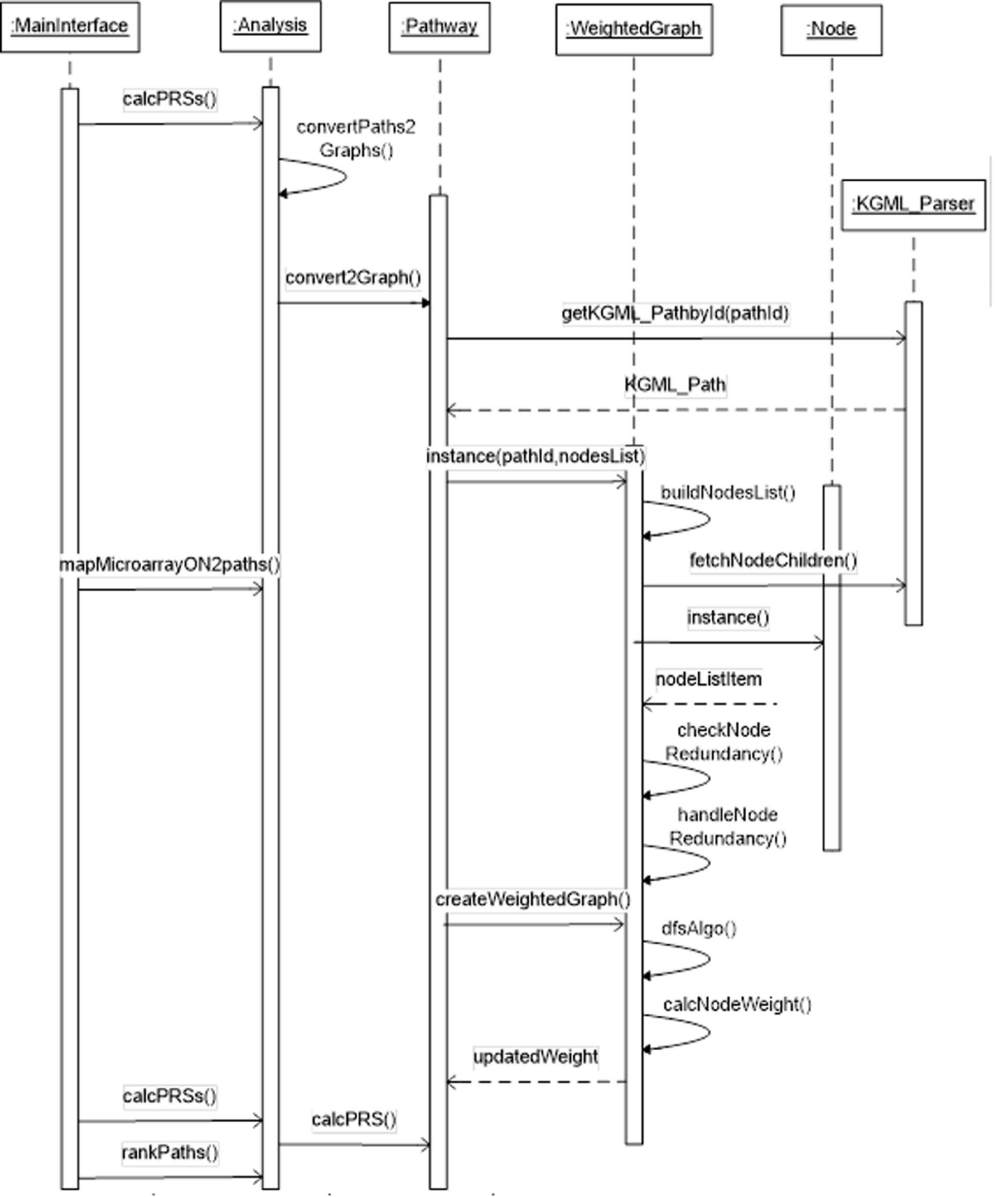
**UML sequence diagram illustrating PRS calculation and pathway ranking.**

Sequence diagrams were used to represent the functions of the PRS tool according to different types of interactions between objects. As an example, Figure 5 represents the main PRS functions with the following steps:

i. Conversion of pathways into graphs by the convertPath2Graph() function, which requires the usage of kgmlObject that holds a list of entries, relations and reactions of all pathways.
ii. Using information stored in kgmlObject and PathsList for each graph (see Figure 4), a list of nodes is created (where each node represents one or more genes from the original pathway) and a list of children for each node.
iii. Removal of redundant genes, which may be represented many times in the same pathway. Two functions are designed to deal with node redundancy: checkNodeRedundancy() and handleNodeRedundancy().
iv. After building a graph for each pathway, graphs are weighted by calling the createWeightedGraphs() function, which uses the DFS algorithm to traverse the nodes of each graph and assign a weight for each significant node taking into account the loops in the graph.
v. A pathway regulation score (PRS) is assigned to each weighted graph using the weights of the significant nodes in the graph and other parameters.

We implemented all these classes, functions, and DFS algorithm using MATLAB R2010a.

## Results and discussion

The objective evaluation of novel enrichment analysis methods is difficult, relying on their ability to discern biological processes already known to be perturbed in disease states. We and others previously attempted this by studying performance across a range of datasets derived from distinct conditions ([27] and references therein). Having extended our algorithm to include biochemical pathways, we performed further analysis on a dataset describing a common metabolic disorder, that of type 2 diabetes mellitus (T2DM). The data were originally created by Taneera et al [31], who compared gene expression levels in RNA isolated from human pancreatic islets taken from 9 type 2 diabetes (T2D) cadaver donors with RNA samples of pancreatic islets derived from 54 non-diabetic cadaver donors. These were hybridised to Affymetrix Human Gene 1.0 ST Arrays, and resulting expression values normalised by Robust Multi-array Analysis (RMA) before being uploaded to the Gene Expression Omnibus (www.ncbi.nlm.nih.gov/geo; accession #GDS4337). We created an input file containing Affymetrix probe IDs and normalized gene expression data for each of the 63 samples. Other parameters required were sample numbers in each group (9 in group1, 54 in group2 in this case), and fold-change and p-value threshold values to filter significant genes (in this case fold-change ≥1.3 and p-value <0.05). Fold-change thres-holds are arbitrary, and the value selected in this example yielded a sufficient number of impacted genes to allow pathway mapping (in this example, a threshold of 1.5 would have yielded only 88 DEGs). The user can opt to enrich for signalling or metabolic pathways, or both (as in this example). Additional statistical testing can be performed, if required, by our permutation method (in this example we used number of permutations = 1000 and p-value threshold = 0.05). Tables 1 and 2 display the top ten pathways ranked according to PRS and z-scores respectively, where only significant pathways (FDR < 0.05) were selected. A number of processes relevant to T2DM were picked up by both techniques, notably metabolic pathways such as “Arachidonic acid metabolism” [32] and “Fatty acid metabolism” [33,34], as well as anticipated signalling processes such as “PPAR signalling pathway“[35,36]. Both techniques detect “Pathways in cancer”, which is unsurprising as this description encompasses a number of processes perturbed in diabetes including apoptosis and the cell cycle, along with TGF-beta signalling [37]. “Complement and coagulation cascades” scored highly with both methods, which could be a false positive or may reflect alterations to the vasculature in diabetic islets. Apart from this exception, all other high-scoring PRS pathways are known to be impacted in diabetic states. Conversely, a number of pathways detected by z-scoring are harder to explain, and so may also be false positives (“Intestinal immune network”, “Cell adhesion molecules”, “Allograft rejection”, “Staphylococcus aureus infection”). Finally, the PRS method afforded greater prominence to two pathways critical to T2DM, “MAPK signalling” [38] and “Type II diabetes mellitus” [39], compared to z-scoring. Indeed, the latter description explicitly reflects the impact on adipocytokine and insulin signalling, which are central to the pathophysiology of diabetes.

**Table 1:** **Top ten pathways ranked by PRS (T2D and pancreatic islets dataset)**

**Rank**	**Pathway name**	**PRS**	**pvalue**	**FDR**
1	Arachidonic acid metabolism	3.450412	0	0
2	Cytokine-cytokine receptor interaction	1.443531	0	0
3	TGF-beta signalling pathway	1.345376	0	0
4	Complement and coagulation cascades	1.180362	0	0
5	PPAR signaling pathway	1.030316	0.002	0.0065
6	Pathways in cancer	0.910555	0.004	0.0104
7	Type II diabetes mellitus	0.793327	0.002	0.0065
8	Tryptophan metabolism	0.754089	0.001	0.004875
9	MAPK signaling pathway	0.736616	0.001	0.004875
10	Fatty acid metabolism	0.701842	0.004	0.0104

**Table 2:** **Top ten pathways ranked by Z-score (T2D and pancreatic islets dataset)**

**Rank**	**Pathway**	**Z-score**
1	Arachidonic acid metabolism	6.103672
2	TGF-beta signaling pathway	5.571651
3	Complement and coagulation cascades	5.468563
4	PPAR signaling pathway	5.302763
5	Cytokine-cytokine receptor interaction	5.102405
6	Fatty acid metabolism	5.050608
7	Intestinal immune network for IgA production	4.748036
8	Cell adhesion molecules (CAMs)	4.601507
9	Allograft rejection	4.480696
10	Staphylococcus aureus infection	4.416682

## Conclusions

The rapid development of high-throughput genomic technologies and the deposition of their output in open-access databases has produced huge amounts of biolo-gical data. Mining and interpreting these data has driven innovation in the field of computational biology, leading to the emergence of sophisticated tools to produce reliable, meaningful and testable results. This is important as these kinds of experiments are expensive, and new tools are likely to add value to pre-existing analysis.

In this paper, we address two areas; firstly, the extension of our PRS enrichment algorithm [27] to include both metabolic and signalling pathways; and secondly, to provide a detailed description of a GUI that facilitates array analysis by both PRS and z-scoring. The improved tool handles a number of challenges, notably in ID mapping, redundancy in pathway descriptions and statistical significance assessment. Unlike z-scoring, the PRS algorithm takes into account the topology of a pathway (the relationships between genes) and the magnitude of gene expression changes to identify impacted pathways. For these reasons, we argue that PRS enrichment yields more biologically-relevant insights compared to those provided by the standard hypergeometric method. It was not feasible to compare performance to other PT methods as the additional preprocessing steps taken to reduce redundancy in KEGG descriptions are not easily implemented in other methods without considerable re-engineering. The behaviour of signalling and metabolic pathways is, of course, distinct. However, as our approach was to assess transcriptional changes in a pathway, rather than to predict an effect on the function of a pathway, we felt it was reasonable to evaluate impact on signalling and biochemical pathways using a single method. In this way, we were able to detect biochemical pathways known to be perturbed in metabolic disease. A key tenet of this kind of analysis is that biomedical scientists are guided in the subsequent investigation of targets revealed by transcriptional profiling studies. Unfortunately, there is no unambiguous statistical test that allows investigators to be certain that any pathway highlighted is worthy of further study (and considerable expense). The use of permutation-based approaches are commonly used to determine the likelihood of an enrichment score being achieved by chance, and by adjusting P values by FDR can increase investigators’ confidence that a result is meaningful.

In summary, we suggest that providing researchers with a choice of analysis tools, informed by distinct rationales, will allow evidence to be combined or contrasted in order to facilitate more informed decision making.

## Availability and requirements

**Project name**: PRS_software.

**Project home page**: http://www.buckingham.ac.uk/research/clore-laboratory-diabetes-obesity-and-metabolic-research/staff/maysson-al-haj-ibrahim/prs-tool/.

**Operating system(s)**: Platform independent.

**Programming language**: MATLAB.

**Other requirements**: MATLAB 2010a or higher. If MATLAB is not installed on your PC, you need to install the MCR (Matlab Compiler Runtime) environment first and then run the PRS tool.

**Restrictions for use**: None.

## Additional files

Additional file 1:
**Objects forming KEGG pathways represented in a KGML file.**
Additional file 2:
**UML sequence diagram representing the implementation of the “initialise” microarray function.**
Additional file 3:
**UML sequence diagram of the parse KGML function.**
Additional file 4:
**UML sequence diagram representing the implementation details of the process of creating the list of pathways from KEGG and mapping microarray data onto them.**

## References

[1] Glazko GV, Emmert-Streib F (2009). Unite and conquer: univariate and multivariate approaches for finding differentially expressed gene sets. Bioinformatics.

[2] Khatri P, Sirota M, Butte AJ (2012). Ten Years of Pathway Analysis: Current Approaches and Outstanding Challenges. PLoS Comput Biol.

[3] Dahlquist KD, Salomonis N, Vranizan K, Lawlor SC, Conklin BR (2002). GenMAPP, a new tool for viewing and analyzing microarray data on biological pathways. Nat Genet.

[4] Zeeberg BR, Feng W, Wang G, Wang MD, Fojo AT, Sunshine M, Narasimhan S, Kane DW, Reinhold WC, Lababidi S, Bussey KJ, Riss J, Barrett JC, Weinstein JN (2003). GoMiner: a resource for biological interpretation of genomic and proteomic data. Genome Biol.

[5] Khatri P, Draghici S, Ostermeier GC, Krawetz SA (2002). Profiling Gene Expression Using Onto-Express. Genomics.

[6] Al-Shahrour F, Díaz-Uriarte R, Dopazo J (2004). FatiGO: a web tool for finding significant associations of Gene Ontology terms with groups of genes. Bioinformatics.

[7] Subramanian A, Tamayo P, Mootha VK, Mukherjee S, Ebert BL, Gillette MA, Paulovich A, Pomeroy SL, Golub TR, Lander ES, Mesirov JP (2005). Gene set enrichment analysis: a knowledge-based approach for interpreting genome-wide expression profiles. Proc Natl Acad Sci U S A.

[8] Tian L, Greenberg SA, Kong SW, Altschuler J, Kohane IS, Park PJ (2005). Discovering statistically significant pathways in expression profiling studies. Proc Natl Acad Sci USA.

[9] Barry WT, Nobel AB, Wright FA (2005). Significance analysis of functional categories in gene expression studies: a structured permutation approach. Bioinformatics.

[10] Boorsma A, Foat BC, Vis D, Klis F, Bussemaker HJ (2005). T-profiler: scoring the activity of predefined groups of genes using gene expression data. Nucleic Acids Res.

[11] Kim S-B, Yang S, Kim S-K, Kim SC, Woo HG, Volsky DJ, Kim S-Y, Chu I-S (2007). GAzer: gene set analyzer. Bioinformatics.

[12] Backes C, Keller A, Kuentzer J, Kneissl B, Comtesse N, Elnakady YA, Muller R, Meese E, Lenhof H-P (2007). GeneTrail-advanced gene set enrichment analysis. Nucleic Acids Res.

[13] Mitrea C, Taghavi Z, Bokanizad B, Hanoudi S, Tagett R, Donato M, Voichiţa C, Drăghici S (2013). Methods and approaches in the topology-based analysis of biological pathways. Front Physiol.

[14] MetaCore™: [http://thomsonreuters.com/metacore/]

[15] Glaab E, Baudot A, Krasnogor N, Schneider R, Valencia A (2012). EnrichNet: network-based gene set enrichment analysis. Bioinformatics.

[16] Amin K (2007). Pathway-express: A Bioinformatics Tool for Pathway Level Analysis Using Gene Expression Data.

[17] Fang Z, Tian W, Ji H (2012). A network-based gene-weighting approach for pathway analysis. Cell Res.

[18] Greenblum SI, Efroni S, Schaefer CF, Buetow KH (2011). The PathOlogist: an automated tool for pathway-centric analysis. BMC Bioinformatics.

[19] Jacob L, Neuvial P, Dudoit S (2010). Gains in power from structured two-sample tests of means on graphs.

[20] Mieczkowski J, Swiatek-Machado K, Kaminska B (2012). Identification of Pathway Deregulation – Gene Expression Based Analysis of Consistent Signal Transduction. PLoS One.

[21] Khatri P, Voichita C, Kattan K, Ansari N, Khatri A, Georgescu C, Tarca AL, Draghici S (2007). Onto-Tools: new additions and improvements in 2006. Nucleic Acids Res.

[22] Shojaie A, Michailidis G (2009). Analysis of Gene Sets Based on the Underlying Regulatory Network. J Comput Biol.

[23] Rahnenführer J, Domingues FS, Maydt J, Lengauer T (2004). Calculating the Statistical Significance of Changes in Pathway Activity From Gene Expression Data. Stat Appl Genet Mol Biol.

[24] Gao S, Wang X (2007). TAPPA: topological analysis of pathway phenotype association. Bioinformatics.

[25] Xia J, Wishart DS (2010). MetPA: a web-based metabolomics tool for pathway analysis and visualization. Bioinformatics.

[26] Martini P, Sales G, Massa MS, Chiogna M, Romualdi C (2012). Along signal paths: an empirical gene set approach exploiting pathway topology. Nucleic Acids Res.

[27] Ibrahim MA, Jassim S, Cawthorne MA, Langlands K (2012). A Topology-Based Score for Pathway Enrichment. J Comput Biol.

[28] **Kyoto Encyclopaedia of Genes and Genomes**, data retrieved May 2012 from [http://www.genome.jp/kegg/]

[29] Cheadle C, Vawter MP, Freed WJ, Becker KG (2003). Analysis of Microarray Data Using Z Score Transformation. J Mol Diagn.

[30] Unified Modeling Language™ (UML®): [http://www.uml.org/]

[31] Taneera J, Lang S, Sharma A, Fadista J, Zhou Y, Ahlqvist E, Jonsson A, Lyssenko V, Vikman P, Hansson O, Parikh H, Korsgren O, Soni A, Krus U, Zhang E, Jing X-J, Esguerra JLS, Wollheim CB, Salehi A, Rosengren A, Renström E, Groop L (2012). A Systems Genetics Approach Identifies Genes and Pathways for Type 2 Diabetes in Human Islets. Cell Metab.

[32] Persaud SJ, Muller D, Belin VD, Kitsou-Mylona I, Asare-Anane H, Papadimitriou A, Burns CJ, Huang GC, Amiel SA, Jones PM (2007). The Role of Arachidonic Acid and Its Metabolites in Insulin Secretion From Human Islets of Langerhans. Diabetes.

[33] Yaney GC, Corkey BE (2003). Fatty acid metabolism and insulin secretion in pancreatic beta cells. Diabetologia.

[34] McGarry JD (2002). Banting lecture 2001 Dysregulation of fatty acid metabolism in the etiology of type 2 diabetes. Diabetes.

[35] Sugden MC, Holness MJ (2004). Potential Role of Peroxisome Proliferator-Activated Receptor-α in the Modulation of Glucose-Stimulated Insulin Secretion. Diabetes.

[36] Kim H-S, Hwang Y-C, Koo S-H, Park KS, Lee M-S, Kim K-W, Lee M-K (2013). PPAR-γ Activation Increases Insulin Secretion through the Up-regulation of the Free Fatty Acid Receptor GPR40 in Pancreatic β-Cells. PLoS One.

[37] Prentki M, Nolan CJ (2006). Islet cell failure in type 2 diabetes. J Clin Invest.

[38] Tomas A, Yermen B, Min L, Pessin JE, Halban PA (2006). Regulation of pancreatic β-cell insulin secretion by actin cytoskeleton remodelling role of gelsolin and cooperation with the MAPK signalling pathway. J Cell Sci.

[39] Tanizawa Y, Riggs AC, Chiu KC, Janssen RC, Bell DS, Go RPC, Roseman JM, Acton MT, Permutt MA (1994). Variability of the pancreatic islet beta cell/liver (GLUT 2) glucose transporter gene in NIDDM patients. Diabetologia.

