# Supplementary material for: A MATLAB tool for pathway enrichment using a topology-based pathway regulation score
Source: BMC Bioinformatics. 2014 Nov 4;15(1):358. doi: 10.1186/s12859-014-0358-2 (PMC4255424; doi:10.1186/s12859-014-0358-2)
Supplement: Additional file 1: — Objects forming KEGG pathways represented in a KGML file. [file 12859_2014_358_MOESM1_ESM.pdf]

Additional file1

Herein, we describe the objects utilized by KEGG in building the pathways and later by us in building the graphs:

- Entry: this object has a number of attributes including *ID,* a unique positive integer; *Name,* the KEGG ID of the entry; and *Type,* which specifies the type of entry such as "gene" when it is a gene product (usually referring to a protein), or "group" when it is a complex of gene products (a higher order protein complex, or where the same function can be performed by different proteins, for example in different tissue compartments).

- Relation: this object represents the relationship between two proteins or between a protein and compound. It is indicated by an arrow or a line connecting two nodes in a KEGG pathway. It has several attributes including *Entry1* and *Entry2,* which represent the IDs of the two entries that are connected by this relation; *Type,* which indicates the type of connected entries, including protein-protein interaction "PPrel", protein-compound interaction "PCrel", or transcription factor/target interaction "GErel". The relation object might have a sub-object called *subtype,* which gives more details about the nature of the relation and has two attributes; *Name* which specifies the type of connection or relation such as "phosphorylation", "expression", or "inhibition", and *Value,* which gives symbols for the different types of relations (e.g. "+p" and "--|" for "phosphorylation" and "inhibition" respectively).

- Reaction: this object represents a chemical reaction between a substrate and a product represented by an arrow connecting two circles in a KEGG pathway. Each reaction has three attributes: *ID, Name,* and *Type.* The latter represents the direction of a reaction through the values "reversible" or "irreversible". The reaction object has two sub-objects "substrate" and "product" which are identified by their ID and *Name* values.

It is important to note that in KGML, representations of signalling pathways, proteins (boxes) are linked by "relations" whereas in the metabolic pathways, enzymes (boxes) are linked by "relations", and compounds (circles) are linked by "reactions”.

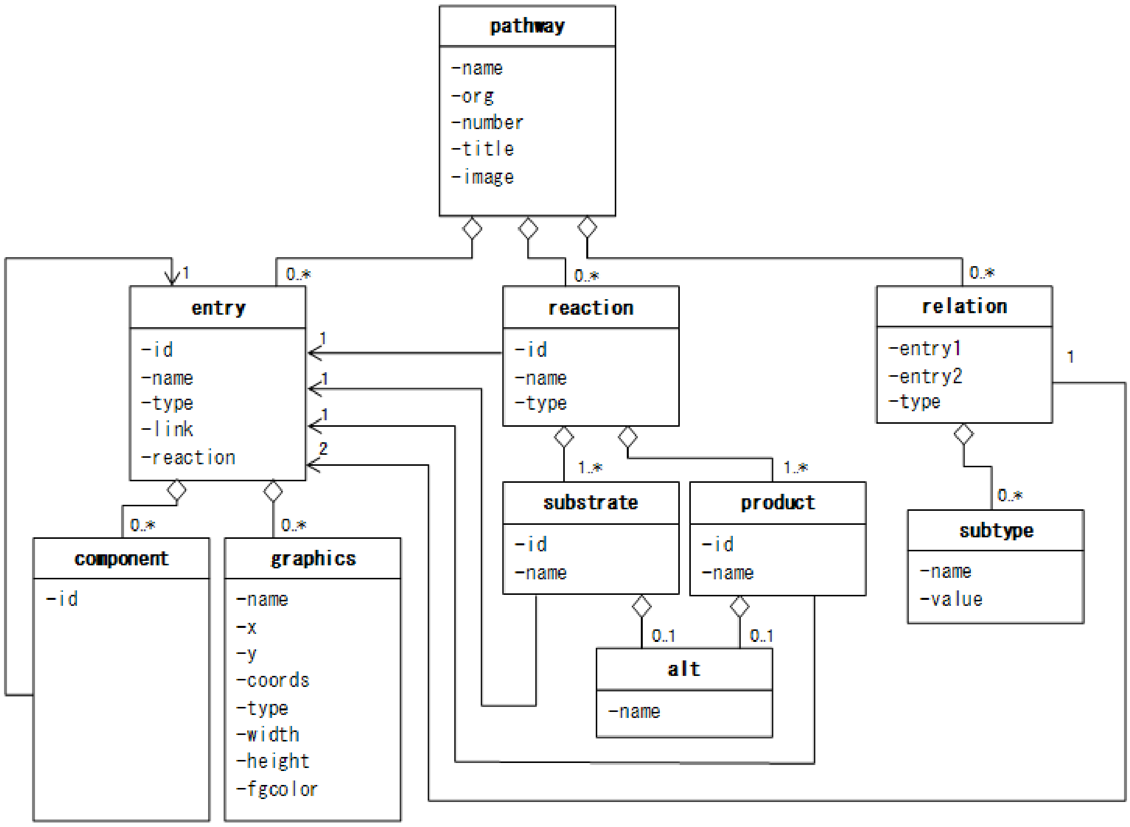

Objects forming KEGG pathways represented in a KGML file (this figure is adapted from http://www.kegg.jp/kegg/xml/docs/)
