# Supplementary figures and images for: A MATLAB tool for pathway enrichment using a topology-based pathway regulation score
Source: BMC Bioinformatics. 2014 Nov 4;15(1):358. doi: 10.1186/s12859-014-0358-2 (PMC4255424; doi:10.1186/s12859-014-0358-2)

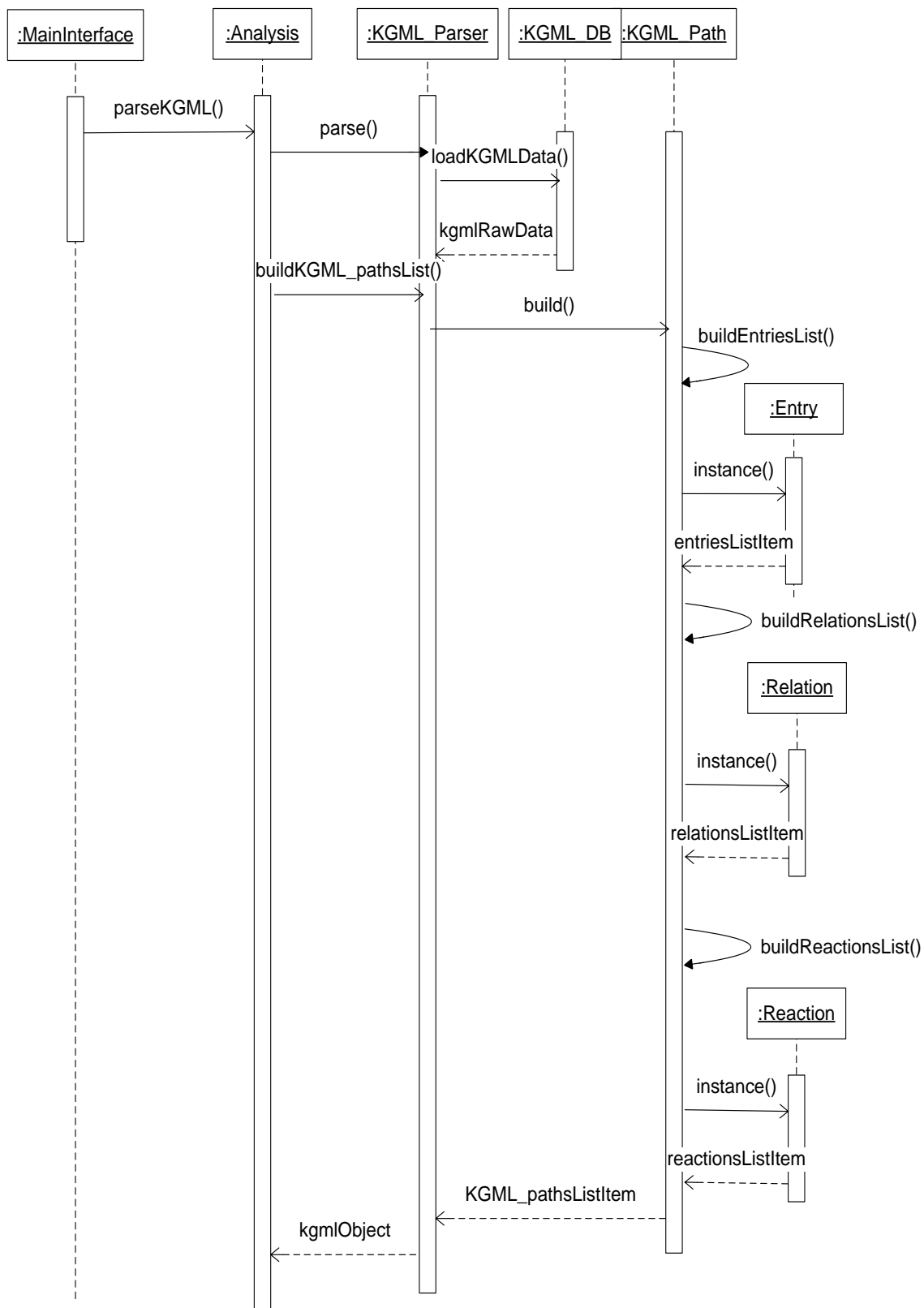

UML sequence diagram of the parse KGML function.

Supplement: Additional file 3: — UML sequence diagram of the parse KGML function. [file 12859_2014_358_MOESM3_ESM.pdf]
