# Supplementary material for: A MATLAB tool for pathway enrichment using a topology-based pathway regulation score
Source: BMC Bioinformatics. 2014 Nov 4;15(1):358. doi: 10.1186/s12859-014-0358-2 (PMC4255424; doi:10.1186/s12859-014-0358-2)
Supplement: Additional file 4: — UML sequence diagram representing the implementation details of the process of creating the list of pathways from KEGG and mapping microarray data onto them. [file 12859_2014_358_MOESM4_ESM.pdf]

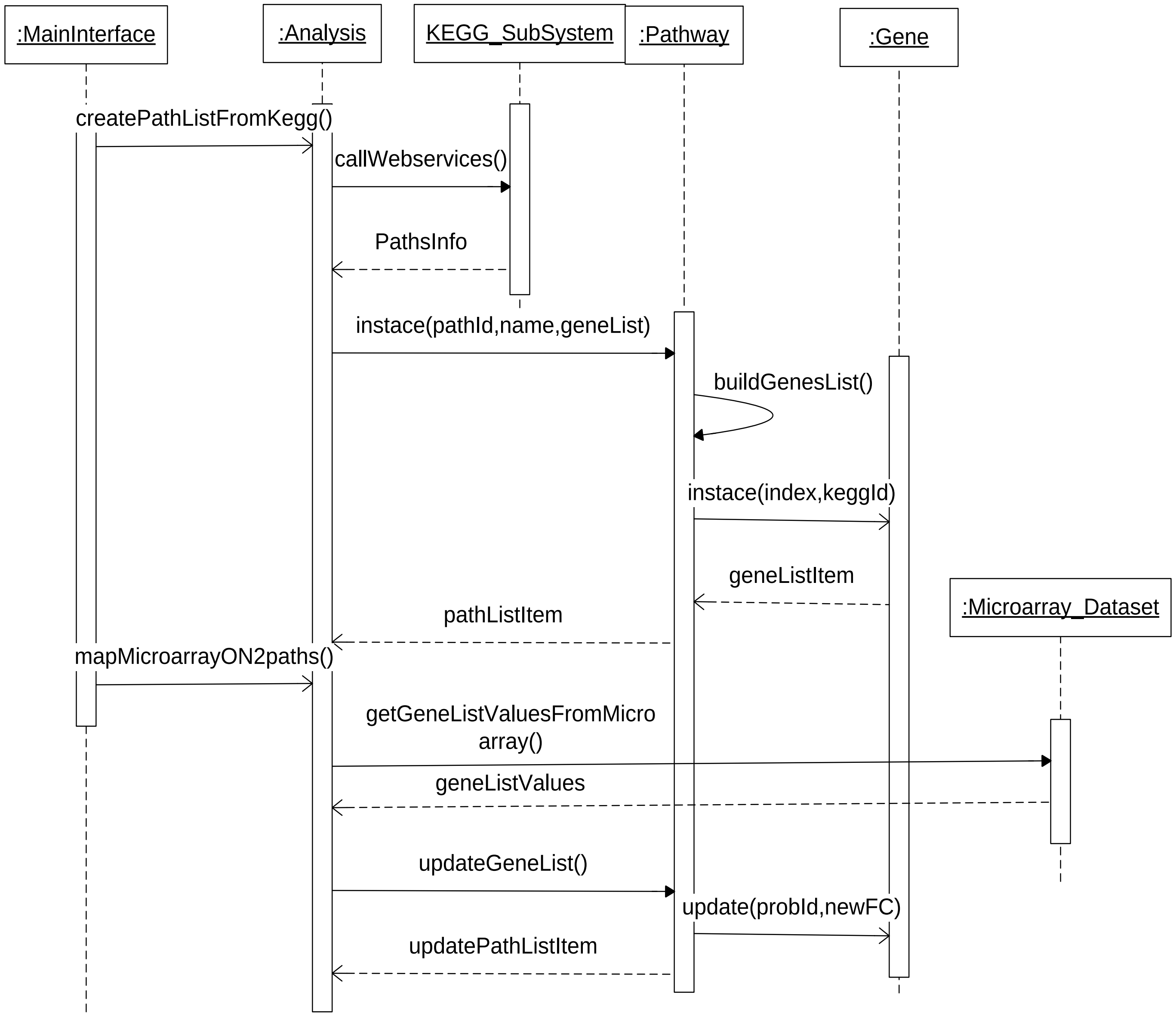

UML sequence diagram representing the implementation details of the process of creating the list of pathways from KEGG and mapping microarray data onto them.
